# Yeast Gene *ILM1* Influences Passage Through G1/S

**DOI:** 10.17912/micropub.biology.002258

**Published:** 2026-07-16

**Authors:** Drew Dewitt, Alyssa Amyx, Kaitlynn Holderman, Carmen Maxwell, Ian Ball, Dylan Bennington, Rilee Terry, Dafne Ruiz Garcia, Rudy Branstetter, Wesley Boyd, Jill Kuglin Schweitzer

**Affiliations:** 1 Department of Natural Science, Indiana University East, Richmond, IN

## Abstract

In this study, *ILM1* , a gene of unknown function, was studied in the yeast *Saccharomyces cerevisiae* . We investigated the sensitivity of an *ILM1* deletion strain to a variety of stress conditions and compared the budding indices of wild-type and *ilm1Δ* strains. Our results showed that deletion of *ILM1* increases sensitivity to caffeine and leads to a decrease in cell budding. These findings are consistent with a new role for *ILM1* in regulating passage through the G1/S transition.

**Figure 1. Deletion of ILM1 sensitizes yeast to caffeine and decreases budding:** **A,** Tenfold serial dilution spot assays of paired wild-type and *ilm1Δ* , spotted on YPD media and 10mM caffeine incubated at 30°C for 2 days. Images show that *ilm1Δ* exhibit sensitivity to caffeine as compared to wild type strain. **B,** Independent spot assays of wild-type and *ilm1Δ* strains on YPG or YPD with 100mM CaCl _2_ or 5% ethanol. Cells were incubated at 30°C for 2-3 days. The growth of wild-type (top three rows) and *ilm1Δ* (bottom three rows) was similar under these stress conditions. **C,** Cell morphology analysis of fixed log-phase cells of both wild-type (left) and *ilm1Δ* (right). Micrographs show an increase in unbudded cells in the *ilm1Δ* strain. Scale bar = 1µm **. D,** Bar graph showing percent unbudded cells of both wild-type and *ilm1Δ* in log-phase cultures (average of three independent trials; 100 cells of each strain counted per trial). The *ilm1Δ* strain has a significantly higher percentage of unbudded cells than wild-type. Wild type: 53% ±8.1%; *ilm1Δ* : 74% ±8.5% (p=0.005).

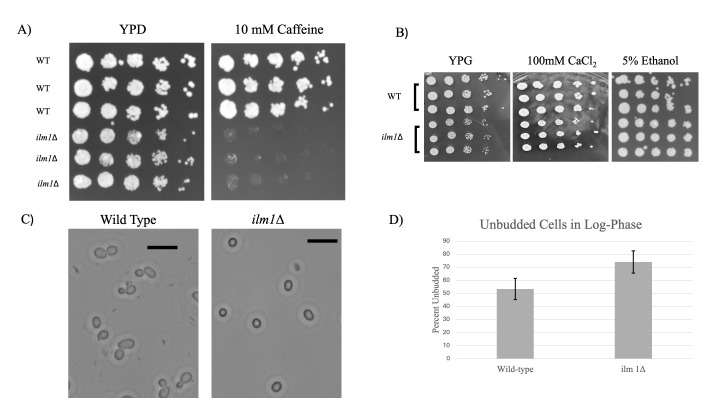



## Description

The function of the yeast gene *ILM1* has not been well-characterized, but it may play a role in mitochondrial DNA maintenance (Entian et al., 1999) and cell wall integrity (Lockshon et al., 2007). The Ilm1p likely localizes to the endoplasmic reticulum (Huh et al., 2003) and only contains one functional protein domain, named *Increased loss of mitochondrial DNA protein 1* (Engel et al., 2025). Based on analysis of *ILM1* interacting genes using GeneMania and SPELL (Hibbs et al., 2007; Warde-Farley et al., 2010), a potential function for *ILM1* in protein trafficking was identified. For example, GO terms related to endoplasmic reticulum-to-Golgi transport were enriched in the SPELL analysis of *ILM1* , and *ILM1* interacts with genes involved in these processes, such as *ERP2* and *GET1* .

To learn more about the potential function of *ILM1* in yeast, a knock-out strain ( *ilm1Δ* ) was made using a PCR-based strategy that replaced *ILM1* with a functional copy of *URA3* in the BY4741 background strain of *S. cerevisiae* . Next, wild-type and *ilm1Δ* strains were grown using spot assays to test a variety of environmental stress conditions. Both wild-type and *ilm1Δ* produced the expected number of colonies on YPD media, but the *ilm1Δ* strain grew more slowly, taking longer to produce large colonies ( Figure 1A ). This finding is consistent with reduced colony size observed for tetrads produced by *ilm1/ILM1* diploids (Entian et al., 1999). When incubated on media containing 5% ethanol or 100mM CaCl _2_ , growth of *ilm1Δ* was like that of wild-type ( Figure 1B ). Also, *ilm1Δ* grew similarly to wild-type on YPG media ( Figure 1B ), suggesting that *ILM1* is not required for mitochondrial function during respiratory growth and consistent with previous work (Entian et al., 1999). However, on YPD containing 10mM caffeine, the growth of the *ilm1Δ* strain was markedly inhibited compared to wild-type ( Figure 1A ). The *ilm1Δ* strain exhibited a hypersensitivity to caffeine.

Caffeine treatment has pleiotropic effects in yeast, including a disruption of the cell wall integrity (CWI) pathway and an inhibition of the TOR signaling pathway that regulates cell growth in response to environmental cues (Ruta & Farcasanu, 2020). Since *ilm1Δ* exhibited slowed growth on YPD and hypersensitivity to caffeine, its budding profile was investigated using morphological analysis. During log-phase growth in YPD, *ilm1Δ* accumulated more unbudded cells than wild-type ( Figure 1C, D). The average percentage of unbudded cells increased from 53% in wild-type to 74% in *ilm1Δ* (p=0.005; t-test comparing results from three independent trials). This result shows that *ilm1Δ* cells appear to have a mild but measurable G1 delay, consistent with their slower growth observed on YPD media. Loss of *ILM1* alone delays G1 progression and this is exacerbated in the presence of caffeine. Our findings suggest that the marked caffeine sensitivity of *ilm1Δ* cells may be due to its inhibition of TORC1.

The TORC1-Sch9 signaling pathway has been shown to regulate lifespan in *S. cerevisiae* and entry into S phase (Deprez et al., 2018; Moreno-Torres et al., 2015). In response to nutrient availability, TORC1 signaling promotes events that support cell division, including ribosome biogenesis and translation initiation (Wei & Zheng, 2011). Our findings show that loss of *ILM1* delays G1 progression and sensitizes cells to TORC1 inhibition by caffeine. Since *ILM1* is required for robust passage through G1/S, it may be acting in parallel with known TORC1 effectors to support efficient growth-dependent initiation of S phase.

## Methods

The methods for this work were adapted from procedures made available through the Yeast ORFan Project (Miller et al., 2024).

**Yeast culture conditions**

Yeast cells were grown at 30ºC in prepared YPD medium (1% yeast extract, 2% peptone, 2% dextrose) in liquid cultures or on solid medium with 2% agar. After yeast transformation, cells were incubated on SD-Ura agar plates for 4-5 days to select for successful transformants. Selection media (SD-Ura) contained 0.67% yeast nitrogen base without amino acids and 2% dextrose, supplemented with 20mg/L l-histidine, 20mg/L l-methionine, and 60mg/L l-leucine. Components for yeast media were from Dot Scientific, Inc., Burton, MI.

***ILM1* knockout**

An *ILM1* knockout strain ( *ilm1Δ* ) was created in the BY4741 *Saccharomyces cerevisiae* background using a PCR-based strategy. First, plasmid DNA containing wild-type *URA3* (pRS406) was isolated from *E. coli* using Qiagen mini-prep kit and quantitated using a NanoDrop 2000 spectrophotometer (ThermoScientific). Next, amplification of *URA3* was accomplished using a forward primer to recognize twenty bases at the beginning of *URA3* with an additional 60 bases of genomic DNA upstream of *ILM1* and a reverse primer to recognize twenty bases at the end of *URA3* plus an additional 60 bases of genomic DNA downstream of *ILM1* . See Table 2 for primer sequences. PCR reactions contained 0.5mM each primer, 0.2 mM dNTPs, 1X PCR buffer (GenScript), 0.5ml GenScript Green Taq DNA polymerase, and approximately 10 ng of plasmid template. Finally, the *URA3* PCR product was introduced into yeast using yeast transformation (Gietz & Schiestl, 2007). Successful deletion strains were confirmed by isolating DNA from transformant colonies (Lõoke et al., 2011) and amplifying a confirmation PCR product—forward primer annealed to the genome upstream of *ILM1* and the reverse primer annealed to *URA3* . All PCR products were analyzed using 0.8% agarose gel electrophoresis.

**Spot assay**

Liquid cultures of wild type and deletion strains were grown overnight in YPD with shaking, diluted to OD _600_ of 0.1 and then serially diluted 1:10 to make five cell suspensions ranging from 1:10 ^0^ to 1:10 ^4^ . Each strain was diluted in triplicate per experiment. 2μl of each cell suspension was spotted onto YPD agar plates and YPD containing 10mM caffeine, 100mM CaCl _2_ , or 5% ethanol (Sigma Aldrich). Cell suspensions were also plated on media containing glycerol instead of dextrose (YPG). Plates were imaged after 2 or 3 days of incubation at 30 ºC. Each spot assay was replicated independently three times and representative images are shown.

**Morphological Analysis**

To prepare log-phase cells, liquid cultures of wild type and deletion strains were grown overnight in YPD with shaking, diluted to OD _600_ of 0.1, and then incubated in fresh YPD for 4-5 hours. Cells were washed with phosphate-buffered saline (1X PBS), fixed with 3.7% formaldehyde for 5 min and sonicated for 20s to reduce clumping using a Fisher Scientific FB505 (amplitude—20%; pulse 1s on/1s off). Sonicated cells were mounted on slides and imaged using brightfield microscopy (Nikon Eclipse Ei with Digital Sight 1000 Camera). Micrographs were analyzed using Image J to measure the area of one hundred cells of each strain per experiment and determine the budding state. Morphological analysis was replicated independently three times. The unbudded percentage shown in Figure 1D is an average of three independent trials. A student t-test was used to compare the number of unbudded cells from wild-type and *ilm1Δ* to determine statistical significance.

## Reagents

**Table 1. Yeast Strains**

**Table:** 

**Name**	**Genotype**	**Source**
BY4741 (wild-type)	*MATa his3Δ1 leu2Δ0 met15Δ0 ura3Δ0*	Horizon Discovery
*ilm1Δ*	*MATa his3Δ1 leu2Δ0 met15Δ0 ura3Δ0 ilm1::URA3*	This study

**Table 2. PCR Primers**

**Table:** 

Knockout of *ILM1* —Amplification of *URA3*	
Forward Primer: 5’ACAATTTACAACATAATAGAGTATCGCATTCAGCAAAAGTAAAGAATAAATTCTAAGAAAATGTCGAAAGCTACATATAAGG-3’	
Reverse Primer: 5’ATACATACACAGGTATCTACTATAAGAATAAAGGAAAGAAAAAATAAACGATTAAACATTTTAGTTTTGCTGGCCGCATC-3’	
Knockout of *ILM1* —Confirmation of *ILM1* disruption with *URA3*	
Forward Primer 5’-GTTCAATAGCCTTTGTATTGCG-3’	Reverse Primer 5’-AAACCGCTAACAATACCTGGG-3’
